# Reconstructing the silent circulation of West Nile Virus in a Caribbean island during 15 years using sentinel serological data

**DOI:** 10.1371/journal.pntd.0012895

**Published:** 2025-06-23

**Authors:** Celia Hamouche, Jennifer Pradel, Nonito Pagès, Véronique Chevalier, Sylvie Lecollinet, Jonathan Bastard, Benoit Durand

**Affiliations:** 1 EPIMIM, Laboratoire de Santé Animale, ANSES, Ecole Nationale Vétérinaire d’Alfort, Maisons-Alfort, France; 2 UMR ASTRE, CIRAD, INRAe, Université de Montpellier, Montpellier, France; 3 ASTRE, CIRAD, Petit-Bourg, Guadeloupe, France; 4 ASTRE, CIRAD, Antananarivo, Madagascar; 5 Sorbonne Université, INSERM, IPLESP, Paris, France; North South University, BANGLADESH

## Abstract

The dynamics of zoonotic infectious diseases with silent circulation may be imperfectly understood and monitored using passive (or reactive) epidemiological surveillance data only, highlighting the interest of quantitative methods like modelling. West Nile virus (WNV) is a widespread mosquito-borne virus transmitted from birds to “dead-end” hosts including humans and horses, in whom it can be fatal. It was first detected in Guadeloupe, Caribbean, in 2002, although no WNV clinical case in humans nor horses had been reported on the archipelago before 2024. Undetected infections represent a risk as WNV can be transmitted *via* blood and organ donations. In Guadeloupe, epidemiological surveillance started in 2002 in chickens and horses and in 2015 in mosquitoes, to detect WNV and to improve knowledge on its epidemiology and dynamics. In order to reconstruct the WNV force of infection (FOI), we built a model assessing different hypotheses regarding its dynamics using serological results in respectively 1,022 and 3,649 blood samples collected from 256 horses and 317 chickens between 2002 and 2018. We fitted the model to the serological data using a Markov Chain Monte Carlo algorithm. We found that WNV FOI in Guadeloupe Island presented both within-year (seasonal) and between-years fluctuations. We identified three main episodes of WNV circulation on the island between 2002 and 2017. During years with circulation, the FOI was predicted to be highest around the months of October-November, although transmission could occur all year long. We estimated a very low weekly seroreversion rate, which is consistent with a lifelong persistence of WNV IgG antibodies in many infected individuals. To conclude, combining longitudinal serological data to a mathematical model allowed reconstructing the recurrent and silent circulation of WNV in this Caribbean island, which could improve surveillance design for better virus detection.

## Author summary

West Nile virus (WNV) is a mosquito-borne virus that can infect birds, humans, and horses. While birds are the main hosts, humans and horses are considered “dead-end” hosts, meaning they cannot further transmit the virus to mosquitoes. In Guadeloupe, WNV was first detected in 2002, but no clinical cases were reported in humans or horses until 2024. Because infections can go unnoticed yet still pose risks – such as transmission through human blood donations – understanding how the virus circulates silently is important. To better characterize WNV circulation, we analyzed over 4,600 blood samples collected from chickens and horses between 2002 and 2018. Using mathematical modeling and serological data, we reconstructed the virus force of infection, the rate at which individuals became infected. The results revealed both seasonal and year-to-year variations, with peaks in transmission around October–November. We identified three significant periods of virus activity. Infected animals tended to retain antibodies for a long time, indicating long-term immunity. This study shows how combining field data and modeling can reveal hidden patterns of viral circulation. It highlights the value of active surveillance and quantitative tools for detecting and managing zoonotic diseases like WNV, especially in regions where they circulate silently.

## Introduction

Sentinel (active or proactive) surveillance is defined as the repeated collection of information from same selected individuals or groups to identify changes in the health status of a specified population over time [1]. It is complementary to passive (reactive or clinical) surveillance designs where health adverse events are reported by stakeholders (e.g., hospitals, veterinarians, …) as part of their usual activities [2–4]. Sentinels may also specifically refer to animals that are periodically monitored and positioned nearby human populations for the surveillance of human health hazards [5]. Although it often requires substantial resources, active surveillance has the advantage to provide a less biased and more complete picture of an infection occurrence [3,6]. It is particularly useful for pathogens that are under-reported by passive surveillance, for instance when asymptomatic infections are frequent as with arboviruses [7,8] or in settings with limited routine surveillance capabilities [9]. Active surveillance is also adapted to zoonotic diseases arising from wildlife, because human infections then result from incidental transmissions from an animal reservoir source with generally less known demographic (movements, interactions between individuals and populations) and epidemiological (pathogen prevalence and mortality) patterns [10]. When such wildlife zoonotic pathogens circulate endemically, it then becomes appropriate to monitor infections in sentinels from better-followed populations such as domestic animals [11–13].

West Nile virus (WNV), an *Orthoflavivirus* transmitted by mosquitoes mostly of the *Culex* genus, meets most of these criteria. Indeed, wild birds are primary WNV reservoirs, although the virus can spread to mammals including horses and humans [14]. In both species, WNV infection is most often asymptomatic but may result in febrile forms (dengue-like symptoms in humans) and, in some cases, in severe neurological symptoms sometimes leading to death. These species are considered “dead-end hosts” since biting mosquitoes cannot get infected after feeding on them nor further transmit the virus [14,15]. However, WNV can still spread among humans through blood transfusions and organ transplantations from asymptomatic infected donors [16,17]. It is therefore of interest for both human and animal health to monitor its circulation over time and space. This is why simultaneous multi-host surveillance of this pathogen has been emphasized [7,18]. Indeed, WNV sentinel surveillance in many countries has been implemented in multiple host species such as horses, wild and domestic birds or zoo animals, and in vectors [19–35].

In the Americas, WNV was first reported in New York (United States) in 1999, and subsequently spread to the rest of North America, Latin America and the Caribbean [36,37]. Guadeloupe archipelago (French West Indies, Caribbean) has a tropical climate and is populated by ~384,000 inhabitants. WNV circulation in this island was first documented in 2002 when anti-WNV antibodies were found in horses [38]. Following this discovery, a surveillance program was implemented in humans, horses, chickens and mosquitoes using several designs, namely serosurveys, active, sentinel (including based on risk areas) and passive surveillance [39]. Although no clinical case in humans nor horses was reported on the archipelago until 2024 [40,41], anti-WNV antibodies were occasionally detected in horses and chickens throughout two decades [39,42], suggesting its silent circulation.

Mathematical and statistical models may allow inferring the dynamics of pathogens’ force of infection in both competent and incidental hosts, using serological data as markers of past infection [43–46]. Such models were used to infer on transmission patterns of other mosquito-borne viruses, such as Zika, Japanese Encephalitis, Dengue or Chikungunya viruses [47–53]. Previous studies also fitted or validated mechanistic models of WNV transmission to serological data [54–56], although not using more than two years of data.

Here, our objective was to quantify the level of silent circulation of WNV in Guadeloupe between 2002 and 2017. We developed a Bayesian model fitted to longitudinal serological data collected in sentinel chickens and horses, to reconstruct both within-year (seasonal) and between-years variations in the WNV force of infection and to estimate key parameters of its epidemiology and testing.

## Materials and methods

### Ethics statement

Animal samplings have been performed following guidelines and legislation applicable to the surveillance of animal and public health risks (Regulation (EU) 2016/429 of the European Parliament and of the Council of 9 March 2016 on transmissible animal diseases and amending and repealing certain acts in the area of animal health (‘Animal Health Law’)); they have been performed by veterinarians with sanitary authorizations upon request of the veterinary services (DAAF971).

### Serological surveillance data

**Fig 1:**
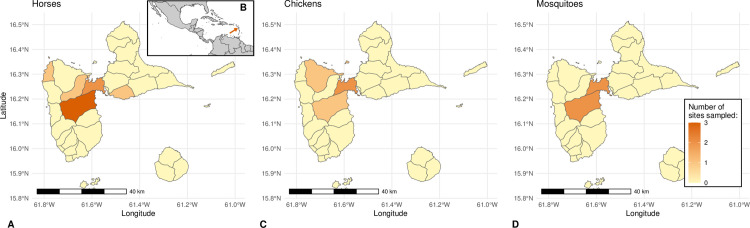
Study sites in Guadeloupe archipelago (Caribbean). Panels A, C and D represent the number of sampling sites per commune, respectively in equine, chicken and mosquito populations. The location was missing for two horse sampling sites (representing 8 out of 1,022 samples). Panel B represents the location of Guadeloupe archipelago in the Caribbean. The base layer maps for this figure were obtained from GADM (https://gadm.org/license.html*) and* geoBoundaries (https://www.geoboundaries.org/*).*

For this study, we analyzed longitudinal serological data collected between 2002 and 2018 in domestic animals in Guadeloupe (Fig 1). Because no clinical case was detected on the archipelago before 2024, the sampling in our study was not driven by animal sickness. Following the introduction of WNV in the Caribbean, almost exhaustive serosurveys were carried out in Guadeloupe horses (2002–2004) [38,42]. Then, a sentinel surveillance scheme was implemented, with horses (starting in 2005) and chickens (starting in 2013) sampled repeatedly in sites assumed to be at higher risk for viral circulation based on a previous study [57]. Finally, we also performed more extended serosurveys following the detection of anti-WNV antibodies in sentinel sites. Our serological dataset collated these multiple surveys. Identifiers associated with each sample allowed us to reconstruct the sequence of serological results for each animal, and individuals with only one result were discarded from the analysis. Some horses moved within Guadeloupe during the period of the study, as part of activities related to the equine industry (e.g., horse riding tours or competitions) or because they changed stable.

**Fig 2:**
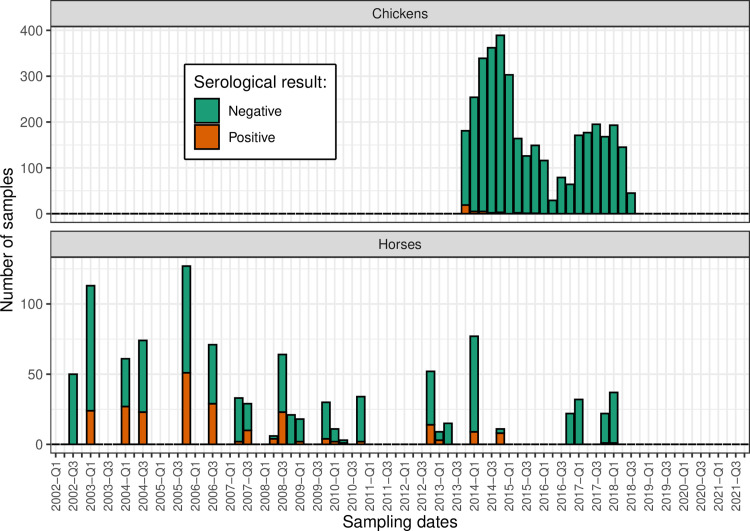
Longitudinal serological data (anti-WNV IgG antibodies) collected from horses and chickens in Guadeloupe between July 2002 and August 2018. Data is represented aggregated by quarter and the x-axis scale is standardized with S4 Fig.

Overall, WNV serological statuses were determined from 1,022 sera sampled from 256 horses in 10 equine centers between July 2002 and February 2018, and from 3,649 sera sampled from 317 chickens in four chicken farms between November 2013 and August 2018 (Figs 1 and 2). The median number of samples per individual was 3 (interquartile range [2; 5]) in horses and 7 (IQR [2; 18]) in chickens (S1 Fig).

Anti-WNV IgG antibodies were detected in sera using inhibition or competition enzyme-linked immunosorbent assays (Epitope Blocking ELISA, targeting respectively anti-NS1 and E antibodies) as previously described [38,58,59]. Both ELISA assays were validated for horse and chicken sera. Threshold values defining ELISA-positives were as specified by the manufacturer for the ELISA E commercial kit, or as determined during the development and validation for the ELISA NS1 [60]. Positive samples were then tested by virus neutralization test at the French Reference Laboratory (ANSES).

We did not have the information on animals’ age at sampling to compute their past exposure to the virus. Therefore, we used the time between consecutive samples taken from same individuals (i.e., pairs of samples) to infer the virus’ force of infection (FOI) most likely to explain serological transitions (e.g., seroconversions). The median duration between consecutive samples in same individuals was 376 days, i.e., 54 weeks, in horses (IQR [306; 593]) and 14 days in chickens (IQR [14; 14]). We discarded two pairs of samples separated by more than 5 years, because we could not reasonably exclude that these animals had undergone more than one serological transition during that period (seroconversion followed by a seroreversion, or the opposite).

### Entomological surveillance data

An entomological surveillance program was set up bi-monthly from November 2015 using CDC CO_2_ mosquito traps (John W. Hock Company, Gainesville, FL) at four sites located near sentinel chicken farms to monitor mosquito population abundances [39,61] (Fig 1). The entomological data used in this study was the abundance of *Culex* mosquitoes. To better capture the time dynamics of vector populations, we used all data available even beyond the period studied – hence collected between November 2015 and May 2021.

### Serological model

Our model aimed to predict the true WNV serological status *S*_*i,k*_ (valued 0 and 1 for negative and positive status, respectively) of the *k^th^* sample taken from individual *i*. For any *k* ≥ 2, *S*_*i,k*_ followed a Bernoulli drawing of probability *p*_*i,k*_:


$$S_{i,k}\sim B(p_{i,k}\backslash$$ (1)

$$\begin{array}{c} p_{i,k}=\left\{ \;\;\;\;\begin{array}{c} 1-\exp\left(-\int_{t_{i,k-1}}^{t_{i,k}}\lambda_{i}(t).dt\right)ifS_{i,k-1}=0\\ exp\left(-\mu \left(t_{i,k}-t_{i,k-1}\right)\right)ifS_{i,k-1}=1 \end{array}\right. \end{array}$$ (2)

Where *t*_*i,k*_ was the week when sample *k* in individual *i* was collected, *λ*_*i*_*(t)* was WNV FOI (i.e., the rate at which hosts become infected) that applied to individual *i* on the week *t*, and *μ* was the seroreversion rate. We assumed that *μ* was constant over time and had the same value for horses and chickens. Moreover, we supposed that the FOI could vary within each year between a baseline and a maximum value (peak height). The maximum FOI was assumed to occur on the same week every year but its value depended on the year. The seasonal variations of *λ*_*i*_ were represented by a sinusoid expressed as:


$$\begin{array}{c} \lambda_{i}(t)=\beta_{i}\left[\frac{\Lambda (y(t))}{2}(1-\varepsilon )\left(1+cos\left(\frac{2\pi }{52}(t-\delta )\right)\right)+\varepsilon \Lambda (y(t))\right] \end{array}$$ (3)

Where *β*_*i*_ was the relative risk of WNV infection in an individual *i* compared to a horse individual, with *β*_*i*_ = 1 if individual *i* was a horse, and *β*_*i*_ = *β* if it was a chicken. *y(t)* corresponded to the year of week *t* and **Λ*(y(t))* was the maximum FOI reached on year *y(t)*. *ε* was the fraction of the FOI that did not vary over the year, hence **εΛ*(y(t))* was the baseline FOI reached on year *y(t)*. *δ* was the week of the year when the peak of FOI was reached (Table 1).

**Table 1:** Description of the serological and observation model parameters in the four scenarios. Some parameters were only used in some of the model scenarios.

Parameter (unit)	Description	FlatStable model	SeasoStable model	FlatVary model	SeasoVary model
*β* (no unit)	Relative risk of WNV infection in chickens as compared to horses	Estimated			
*μ* (week^-1^)	Constant seroreversion parameter	Estimated			
***η*** (no unit)	Sensitivity of serological tests	Estimated			
***ψ*** (no unit)	Specificity of serological tests	Estimated			
***NPV***_***1***_ (no unit)	Negative predictive value of the serological result of the first sample	Estimated			
***PPV***_***1***_ (no unit)	Positive predictive value of the serological result of the first sample	Estimated			
***Λ(y(t))*** (week^-1^)	Maximum (peak) value of WNV FOI over year *y(t)*	**Λ*(y(t)) = *Λ** (estimated, same for all years)		Estimated	
***δ*** (week)	Week number of the yearly peak	Unused	Estimated	Unused	Estimated
***ε*** (no unit)	Fraction of WNV FOI that does not vary within years	**ε* = 1*	Estimated	**ε* = 1*	Estimated

N.B.: The negative predictive value is defined as the probability of true negative given a negative test result, and the positive predictive value as the probability of true positive given a positive test result.

**Fig 3:**
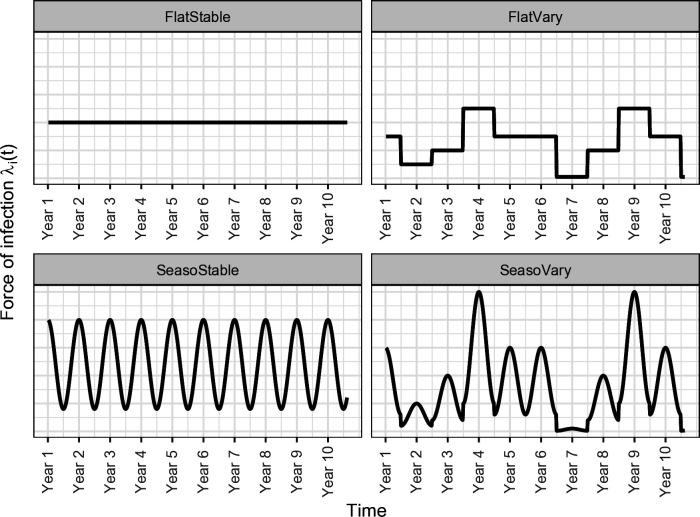
Illustration (synthetic data) of the serological model scenarios used in the study. “SeasoStable” and “FlatVary” models accounted respectively for only within-year (seasonal) and only between-years variations of the force of infection (FOI). “SeasoVary” model accounted for both within- and between-years variations of the FOI. “FlatStable” model did not account for any variation of FOI with time.

We defined four scenarios for the model, depending on whether the FOI varied over time, within and/or between years (Fig 3 and Table 1). In “FlatStable” and “FlatVary” models, *ε* was forced to 1, meaning that the seasonal (i.e., within-year) variations of the FOI were ignored. In “SeasoStable” and “SeasoVary” models, ε was estimated. In “FlatStable” and “SeasoStable” models, **Λ*(y(t)) = *Λ** for all weeks *t*, meaning that we ignored between-year variations. Formulae for each model scenario are summarized in S1 Table.

### Observation model

The serological result *Y*_*i,k*_ (observed WNV serological status) depended on the test sensitivity *η* and specificity *Ψ*, which we both estimated (Table 1). For any sample *k* ≥ 2 in any individual *i*:


$$Y_{i,k}\sim B(\eta .S_{i,k}+(1-\psi ).(1-S_{i,k})\backslash$$ (4)

We did not have the information on animals’ age – hence on their previous exposure to the virus – when they were first sampled. Therefore, in order to initialize the model and infer the true serological status of the first sample in each individual *i*, we introduced parameters *NPV*_*1*_ and *PPV*_*1*_, the negative and positive predictive values of the first sample result for any individual (Table 1). They were respectively defined as the probability of true negative given a first negative test result, i.e., *P(S*_*i,1*_* = 0|Y*_*i,1*_* = 0)*, and as the probability of true positive given a first positive test result, i.e., *P(S*_*i,1*_* = 1|Y*_*i,1*_* = 1)*. *NPV*_*1*_ and *PPV*_*1*_ depended on *η* and *Ψ*, and varied according to hyperparameters as detailed in the S1 Note. For any individual *i*:


$$S_{i,1}\sim B({PPV}_{1}.Y_{i,1}+\left(1-{NPV}_{1}\right).(1-Y_{i,1})$$ (5)

### Models fitting and selection

Model parameters were estimated using a Markov Chain Monte Carlo (MCMC) algorithm, implemented with the R package *rjags* [62]. In this Bayesian framework, most prior distributions were uninformative, although not for parameters *ε* and *δ*. Indeed, we assumed that seasonal variations in WNV FOI (determined by *ε* and *δ*) are partly related to seasonal variations in mosquito abundance. Therefore, we performed the model’s fitting in two steps. In Step 1, we fitted a model analogous to the “SeasoStable” model to the weekly mosquito abundance data, which allowed estimating posterior distributions for seasonality parameters *ε* and *δ* (see details in the S2 Note). These two distributions were then used as informative priors in Step 2, where the four models were fitted to the serological data (S2 Table and S2 Fig). Moreover, in Step 2, we also considered rather informative priors for parameters *η* and *Ψ* using a Beta distribution (S2 Table), because previous publications tended to show a good reliability of serological tests for the detection of WNV antibodies [59,63,64]. In particular, the virus neutralization test is considered the gold standard serological test regarding specificity [59,65]. The usual MCMC convergence diagnostics were performed in both steps. Serological model scenarios were then compared using the Deviance Information Criterion (DIC) and the best fitting model was selected based on the smallest DIC [66].

## Results

### Surveillance results

We analyzed the presence of anti-WNV IgG antibodies in domestic animals sampled repeatedly as part of a sentinel surveillance scheme in Guadeloupe (Figs 1, 2, and S3). Among 764 consecutive pairs of samples collected from horses between 2002 and 2018, 82 (10.7%) seroconversions and 9 (1.2%) seroreversions were observed (Table 2). Among 3,332 consecutive pairs of samples collected from chickens between 2013 and 2018, 6 (0.2%) seroconversions and 6 (0.2%) seroreversions were observed (Table 2).

**Table 2:** Observed serological results (anti-WNV IgG antibodies) in consecutive pairs of samples collected from horses and chickens in Guadeloupe between 2002 and 2018.

		Horses (n = 764 pairs) First sample of the pair		Chickens (n = 3,332 pairs) First sample of the pair	
		Negative	Positive	Negative	Positive
Second sample of the pair	Negative	537	9	3303	6
	Positive	82	136	6	17

*Culex* mosquito abundance data collected bi-monthly between 2015 and 2021 is displayed in S4 Fig. It showed seasonal patterns that were used to derive informative priors for fitting the serological model.

### Model predictions

**Fig 4:**
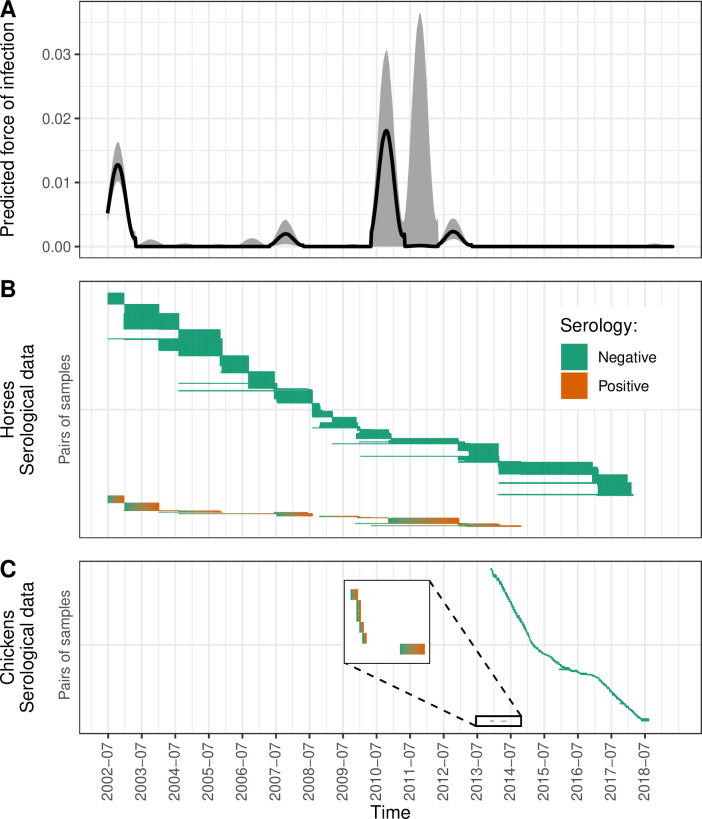
West Nile virus force of infection (FOI) in Guadeloupe predicted between 2002 and 2017 by the “SeasoVary” serological model (panel A), and longitudinal serological data collected in horses (panel B) and chickens (panel C). In panel A, the black line represents the median of predictions (using 5,000 repetitions of the model), while the gray area represents the 80% prediction interval. In panels B and C, each row is a pair of consecutive blood samples, and only the observed negative-to-positive and negative-to-negative serological transitions are displayed.

After fitting the seasonal model to the mosquito abundance data (Step 1), we fitted the four serological models to the longitudinal serological data (Step 2). The model scenario with the lowest DIC was “SeasoVary” (S3 Table), suggesting both within- and between-years variations of WNV FOI in Guadeloupe archipelago. This model predicted that three main episodes of WNV circulation occurred on the island between 2002 and 2017 (see Fig 4): an important one in 2002, followed by another one of smaller intensity in 2007, and finally in 2010–2012, although uncertainty in outbreak intensity (amplitude) was greater for the latter due to less serological data collected. The model scenario with the second lowest DIC was “FlatVary” (ΔDIC = 12, see S3 Table), and also predicted the three same main episodes of WNV circulation (S5 Fig).

### Parameter estimates

Following Step 1 (fit of the seasonal model to the mosquito abundance data), the median of *δ* was estimated to week 45.3 (95% credible interval: [43.5; 47.4]), implying that the yearly peak of *Culex* mosquito abundance in Guadeloupe occurs around October-November (S4 Fig). Then, after Step 2 (fit to serological data) and for the “SeasoVary” model, the median posterior estimate of *δ* was slightly earlier (week 44.9 [42.9; 46.7]), suggesting a marginal time shift for the yearly peak of WNV FOI. Nevertheless, *ε* was estimated to 0.099 [0.023; 0.222], showing the potential of WNV to circulate all year long in Guadeloupe (Table 3 and S6 Fig).

**Table 3:** *Posterior estimates of parameters of the “SeasoVary” serological model: median and 95% highest posterior density interval (HPDI, credible interval). Posterior distributions for* Λ(y(t)) *are depicted in* *S6 Fig*.

Parameter	Unit	Median of the posterior and 95% credible interval (HPDI)
***β***	–	2.63 [0.002; 8.99]
***ε***	–	0.099 [0.023; 0.222]
***δ***	week	44.9 [42.9; 46.7]
***μ***	week^-1^	1.73x10^-3^ [6.08x10^-4^; 2.83x10^-3^]
***η***	–	0.90 [0.769; 0.999]
***ψ***	–	0.999 [0.996; 1.0]
***NPV***_***1***_	–	0.998 [0.995; 1.0]
***PPV***_***1***_	–	0.926 [0.811; 0.998]

We were not able to estimate *β*, the relative risk of infection in chickens as compared to horses, since its posterior distribution (median of 2.63 [0.002; 8.99]) was almost similar to its prior distribution (S6 Fig), reflecting a lack of information in the data regarding this parameter. We estimated the seroreversion parameter to 1.73x10^-3^ [6.08x10^-4^; 2.83x10^-3^] per week, i.e., 1/578 weeks or 1/11.1 years.

Furthermore, we estimated the sensitivity *η* to be 0.90 [0.769; 0.999] and the specificity *ψ* to be 0.999 [0.996; 1.0], confirming the good reliability of serological tests (Table 3 and S6 Fig). *NPV*_*1*_ and *PPV*_*1*_ were estimated to respectively 0.998 [0.995; 1.0] and 0.926 [0.811; 0.998].

What is more, posterior estimates for the “FlatVary” model were similar to the “SeasoVary” model (S7 Fig).

## Discussion

Some infectious diseases that are transmitted to humans from wildlife reservoir sources, such as WNV, may not be well detected by passive surveillance systems, especially when asymptomatic forms are frequent, sparking a possible silent circulation of the pathogen. In this study, we quantified this silent circulation for WNV in Guadeloupe archipelago using a serological model fitted to sentinel surveillance data collected in horses and chickens. We assessed different hypothesis on the variations of WNV force of infection (FOI) by comparing several versions of a phenomenological model that represented over time the rate at which sentinel animals become infected, capturing multiple mechanisms (mosquito abundance, biting rate, density of infectious reservoir hosts, …) all at once. The best selected model was “SeasoVary”, suggesting that the FOI changes both within-year and between-years on the island.

Several ecological and epidemiological mechanisms could explain between-years FOI variations. First, variations of climatic factors across years – including occurrence of extreme weather events – may cause heterogeneity in vectors abundance, species composition and/or competence resulting in dramatic changes of epidemiological patterns [56,67–71]. Specifically, previous studies highlighted a correlation between yearly mosquito abundance and the number of WNV human cases, while others did not or rather put forward vector capacity, partially related to the weather-dependent extrinsic incubation period [69,72–74]. Second, wild bird population renewal over the years may lead to decreasing levels of herd immunity, hence allowing WNV outbreaks to occur again every few years [75]. Third, WNV infection prevalence in migratory birds might vary across years, leading to hypothetical between-years fluctuations in the virus’ introduction risk [76].

Moreover, within-year variations in FOI might also be attributed to various factors. First, we found that the abundance of *Culex* spp. mosquitoes – main WNV vectors – can vary seasonally in such a tropical climate, which was expected [77,78]. Additionally, the distribution of species within the *Culex* genus (including potential enzootic and bridge vectors which are yet to be fully characterized in Guadeloupe) may also change seasonally and may have implications on WNV transmission [79,80]. Second, mechanisms other than mosquito abundance might affect the timing of WNV FOI peak over the year. Indeed, the FOI is also driven by the infection prevalence in vectors, which depends on the seasonal dynamics of infection in wild birds, among other factors. They may depend on demographic traits such as the hatching season, which abruptly supplements the population in susceptible individuals [81]. Furthermore, bird populations on the island fluctuate according to migrations that are highly seasonal, with for instance shorebirds species flying from North America to Guadeloupe archipelago around August-October [82]. Migratory birds might either seasonally introduce the virus, and/or change the total density of susceptible individuals in the island’s wild bird population [54,83–85]. Seasonal patterns were also observed from sentinel chickens in Florida (United States), where seroconversions mostly occurred between July and August [24].

A limitation of this work is that we did not account for spatial heterogeneity of WNV FOI on the island, whereas it may be impacted by environmental risk factors at the local scale [57]. Therefore, we might have overestimated the FOI, especially in years when the sentinel surveillance scheme was implemented in sites considered at higher risk for viral circulation. However, given Guadeloupe surface area (1,628 square kilometers) and the time between successive blood samples in horses (median of 54 weeks), we cannot preclude that individuals were exposed to mosquito bites in unrecorded locations, as part of horse riding tours, competitions or other events related to the equine industry [57], or when changing stable.

Furthermore, we did not directly relate the mosquito abundance measured on the field (between 2015 and 2021) with serological transitions observed in vertebrate sentinels (between 2002 and 2018), because of the small time overlap between sampling periods. Instead, our two-step fitting approach had the advantage to beneficiate from the information present in the mosquito abundance data (in Step 1) for quantifying the seasonality of WNV FOI (in Step 2), while accounting for other potential drivers of these fluctuations, and without the need of concomitant sampling in vectors and hosts. Therefore, our modelling framework was able to detect a hypothetic time lag between the peak in mosquito abundance and the occurrence of infections in incidental hosts, which was previously suggested [86,87]. In our study, we found that the former (estimated from the mosquito trapping data) was close to the time of FOI peak (estimated from the serological data), with overlapping credible intervals. Although this result is not fully comparable to previous works that rather considered the dynamics of infectious mosquitoes [35,72], because mosquito infection prevalence may not be constant within a year [69].

Previous studies showed the frequent persistence of neutralizing antibodies for at least several months or years in birds [88–91], and data is scarce in equids. Here, our estimation of the rate of IgG antibodies loss (seroreversion parameter) suggests a lifelong carriage of such antibodies in many individuals, especially in chickens which have a shorter lifespan, even though seroreversions remain possible. Therefore, these individuals may benefit from a long-term protection against WNV symptoms and contribute to herd immunity. In the absence of published data comparing the seroreversion rates in horses and chickens, we assumed the value was the same for the two species in our model, which might have biased the estimate for each individual species. In the future, it would be informative to quantify this parameter for multiple species by inoculating several individuals with WNV and longitudinally testing the presence of antibodies several months or years later.

The estimation of the relative risk of infection in chickens as compared to horses (*β*) was not conclusive, probably because, in our dataset, the time overlap between sampling periods in horses and in chickens was short and with a low-level WNV circulation. Again, sampling longitudinally both species during an outbreak or as part of an infection experiment would allow assessing this parameter by comparing FOI applying to both species simultaneously. It would be an important metric to consider when comparing different sentinel surveillance strategies, and to prioritize either horses or domestic birds surveillance [92]. At equal *Culex* vector densities in the environment, hosts’ relative risk of infection notably depends on mosquitoes feeding preferences. While *Cx. quinquefasciatus* and *Cx. nigripalpus* are the two main candidate vectors for WNV in Guadeloupe Island [57], the former has been suggested to bite birds (including chickens) more than mammals, whereas it might be the opposite for the latter [79,93–96]. However, host selection have been shown to depend on environmental factors (urban vs. rural settings, hygrometry, etc.) and host availability [79], and they have yet to be fully determined for *Culex* spp. in Guadeloupe. Feeding preferences may also change with host skin surface area availability – which depends on animals’ size – rather than at the host individual level [97].

Our study quantified the regular circulation of WNV in Guadeloupe after 2002, based on sentinel surveillance data, despite no clinical report in humans or horses before 2024. However, it did not allow to determine whether it was due to series of virus introductions or as a result of a local enzootic circulation. Although no blood samples were collected earlier than July 2002, an earlier circulation of WNV on the island cannot be ruled out since a West Nile human case was detected as early as August 2001 in the Cayman Islands, a northern Caribbean territory [98]. From our median estimates of WNV FOI (e.g., in 2007 and 2012), the annual incidence rate in horses during outbreak years can be estimated to 5%-7%. Considering a total estimated equine population between ~500 (in 2003–2004) and ~1,000 (in 2017) on the island [57,99] – which is an overestimation of the susceptible equine population – and a proportion of neurological symptoms of ~10% of WNV infected horses [100,101], we could expect up to ~3–7 equine neurological disease cases in 2007 and 2012. Therefore, the lack of WNV clinical case evidence in both domestic animals and humans until 2024 may suggest a low sensitivity of WNV passive surveillance [39]. Indeed, in both horses and humans in the Caribbean, it may be jeopardized by the frequent co-occurrence of other pathogens with similar pathogenesis (e.g., equine piroplasmosis) and serological cross-reactivity during the diagnosis, especially for circulating flaviviruses such as dengue (DENV) or Zika (ZIKV) viruses [102,103]. This highlights the potential of a complementary sentinel WNV serological surveillance scheme in domestic animals, subject to the results of a more thorough costs-benefits analysis, as well as the importance of cross-sectoral collaborations. In our study, because neutralization test (NT) cross-reactivity remains theoretically possible with DENV and ZIKV, the misattribution of a seroconversion to WNV cannot be excluded. However, in a previous study led in French Polynesia and New Caledonia, no horse showed positive NT results to both WNV and DENV or ZIKV, suggesting a low NT cross-reactivity [103]. Moreover, in our model, we estimated the specificity parameter to be close to 1, which is consistent with the NT being a highly specific test [59,65].

In the future, building a mechanistic model fitted to infection data in vectors and wild birds would allow to unravel the processes underlying temporal changes in WNV FOI [104]. Based on such a model, a simulation study would help to determine what cost-efficient surveillance strategies (involving a seasonal component or not) could be implemented in the Caribbean to monitor WNV emergence or re-emergence [92], and therefore to mitigate its impacts on human and animal health.

## Supporting information

S1 Fig Distribution of the number of sera collected per individual (horse and chicken). (PDF)

S2 Fig Posterior distributions of two parameters following Step 1. (PDF)

S3 Fig Map with proportion of collected samples that were positive to anti-WNV IgG antibodies per commune. The base layer map for this figure was obtained from GADM: https://gadm.org/download_country.html (link to the license information: https://gadm.org/license.html
(PDF)

S4 Fig Mosquito abundance variations in four collection sites in Guadeloupe between November 2015 and May 2021. (PDF)

S5 Fig WNV force of infection in Guadeloupe Island predicted between 2002 and 2017 by the “FlatVary” serological model, and longitudinal serological data in horses and chickens. (PDF)

S6 Fig Prior and posterior distributions of the “SeasoVary” model parameters. (PDF)

S7 Fig Comparison of the parameters’ posterior distributions with the “FlatVary” and “SeasoVary” models. (PDF)

S1 Table Formula of the force of infection depending on the serological model scenario and the species. (PDF)

S2 Table Prior distributions used in Steps 1 and 2 of model fitting. (PDF)

S3 Table Values of the Deviance Information Criterion (DIC) for the different serological model scenarios. (PDF)

S1 Note Definitions of parameters *NPV*_*1*_ and *PPV*_*1*_.
(PDF)

S2 Note Details on the first step of the model fitting.
(PDF)

## References

[1] Hoinville LJ, Alban L, Drewe JA, Gibbens JC, Gustafson L, Häsler B, et al. Proposed terms and concepts for describing and evaluating animal-health surveillance systems. Prev Vet Med. 2013;112(1–2):1–12. doi: 10.1016/j.prevetmed.2013.06.006 23906392

[2] Guberti V, Stancampiano L, Ferrari N. Surveillance, monitoring and survey of wildlife diseases: a public health and conservation approach. 2014. https://air.unimi.it/handle/2434/237277

[3] Racloz V, Griot C, Stärk KDC. Sentinel surveillance systems with special focus on vector-borne diseases. Anim Health Res Rev. 2006;7(1–2):71–9. doi: 10.1017/S1466252307001120 17389055

[4] Chow A, Leo YS. Surveillance of Disease: Overview. In: Quah SR. International Encyclopedia of Public Health. Oxford: Academic Press. 2017;124–38.

[5] Neo JPS, Tan BH. The use of animals as a surveillance tool for monitoring environmental health hazards, human health hazards and bioterrorism. Vet Microbiol. 2017;203. 10.1016/j.vetmic.2017.02.007 PMC7130562 28619165

[6] Murray J, Cohen AL. Infectious Disease Surveillance. Int Encycl Public Health. 2017;222–9.

[7] Braks M, van der Giessen J, Kretzschmar M, van Pelt W, Scholte E-J, Reusken C, et al. Towards an integrated approach in surveillance of vector-borne diseases in Europe. Parasit Vectors. 2011;4:192. doi: 10.1186/1756-3305-4-192 21967706 PMC3199249

[8] Dirlikov E, Ryff KR, Torres-Aponte J, Thomas DL, Perez-Padilla J, Munoz-Jordan J. Update: ongoing Zika virus transmission - Puerto Rico, November 1, 2015-April 14, 2016. MMWR Morb Mortal Wkly Rep. 2016;65(17):451–5. 27149205 10.15585/mmwr.mm6517e2

[9] Leibler JH, Zakhour CM, Gadhoke P, Gaeta JM. Zoonotic and vector-borne infections among urban homeless and marginalized people in the United States and Europe, 1990–2014. Vector-Borne Zoonotic Dis. 2016;16(7):435–44. 27159039 10.1089/vbz.2015.1863

[10] Stallknecht DE. Impediments to wildlife disease surveillance, research, and diagnostics. In: Childs JE, Mackenzie JS, Richt JA. Wildlife and Emerging Zoonotic Diseases: The Biology, Circumstances and Consequences of Cross-Species Transmission. Berlin, Heidelberg: Springer; 2007;315:445–61. doi: 10.1007/978-3-540-70962-6_17 17848074

[11] Leifels M, Khalilur Rahman O, Sam I-C, Cheng D, Chua FJD, Nainani D, et al. The one health perspective to improve environmental surveillance of zoonotic viruses: lessons from COVID-19 and outlook beyond. ISME Commun. 2022;2(1):107. doi: 10.1038/s43705-022-00191-8 36338866 PMC9618154

[12] Anderson DP, Gormley AM, Bosson M, Livingstone PG, Nugent G. Livestock as sentinels for an infectious disease in a sympatric or adjacent-living wildlife reservoir host. Prev Vet Med. 2017;148. 10.1016/j.prevetmed.2017.10.015 29157368

[13] Durand B, Haskouri H, Lowenski S, Vachiery N, Beck C, Lecollinet S. Seroprevalence of West Nile and Usutu viruses in military working horses and dogs, Morocco, 2012: dog as an alternative WNV sentinel species?. Epidemiol Infect. 2016;144(9):1857–64. doi: 10.1017/S095026881600011X 26838515 PMC9150636

[14] Campbell GL, Marfin AA, Lanciotti RS, Gubler DJ. West Nile virus. Lancet Infect Dis. 2002;2(9):519–29. 12206968 10.1016/s1473-3099(02)00368-7

[15] Habarugira G, Suen WW, Hobson-Peters J, Hall RA, Bielefeldt-Ohmann H. West Nile Virus: An Update on Pathobiology, Epidemiology, Diagnostics, Control and “One Health” Implications. Pathogens. 2020;9(7):589. doi: 10.3390/pathogens9070589 32707644 PMC7400489

[16] Pisani G, Cristiano K, Pupella S, Liumbruno GM. West Nile Virus in Europe and Safety of Blood Transfusion. Transfus Med Hemother. 2016;43(3):158–67. doi: 10.1159/000446219 27403087 PMC4924479

[17] Čabanová V, Kerlik J, Kirschner P, Rosochová J, Klempa B, Sláviková M, et al. Co-Circulation of West Nile, Usutu, and Tick-Borne Encephalitis Viruses in the Same Area: A Great Challenge for Diagnostic and Blood and Organ Safety. Viruses. 2023;15(2):366. doi: 10.3390/v15020366 36851580 PMC9966648

[18] Gossner CM, Marrama L, Carson M, Allerberger F, Calistri P, Dilaveris D. West Nile virus surveillance in Europe: moving towards an integrated animal-human-vector approach. Eurosurveillance. 2017;22(18):30526. 28494844 10.2807/1560-7917.ES.2017.22.18.30526 PMC5434877

[19] Mattar S, Komar N, Young G, Alvarez J, Gonzalez M. Seroconversion for West Nile and St. Louis encephalitis viruses among sentinel horses in Colombia. Mem Inst Oswaldo Cruz. 2011;106(8):976–9. doi: 10.1590/s0074-02762011000800012 22241119

[20] Jiménez-Clavero MA, Llorente F, Sotelo E, Soriguer R, Gómez-Tejedor C, Figuerola J. West Nile virus serosurveillance in horses in Donana, Spain, 2005 to 2008. Vet Rec. 2010;167(10):379–80. doi: 10.1136/vr.c3155 20817900

[21] Folly AJ, Waller ESL, McCracken F, McElhinney LM, Roberts H, Johnson N. Equine seroprevalence of West Nile virus antibodies in the UK in 2019. Parasit Vectors. 2020;13(1):596. 33243297 10.1186/s13071-020-04481-9 PMC7690108

[22] Petrović T, Šekler M, Petrić D, Vidanović D, Debeljak Z, Lazić G, et al. Intensive West Nile Virus Circulation in Serbia in 2018-Results of Integrated Surveillance Program. Pathogens. 2021;10(10):1294. doi: 10.3390/pathogens10101294 34684243 PMC8540029

[23] Tamba M, Bonilauri P, Galletti G, Casadei G, Santi A, Rossi A, et al. West Nile virus surveillance using sentinel birds: results of eleven years of testing in corvids in a region of northern Italy. Front Vet Sci. 2024;11. 10.3389/fvets.2024.1407271 PMC11138491 38818494

[24] Riles MT, Martin D, Mulla C, Summers E, Duke L, Clauson J. West Nile Virus Surveillance in Sentinel Chickens and Mosquitoes in Panama City Beach, Florida, from 2014 to 2020. J Am Mosq Control Assoc. 2022;38(3):148–58. 35925833 10.2987/22-7074

[25] Chaskopoulou A, Dovas CI, Chaintoutis SC, Kashefi J, Koehler P, Papanastassopoulou M. Detection and early warning of West Nile Virus circulation in Central Macedonia, Greece, using sentinel chickens and mosquitoes. Vector Borne Zoonotic Dis. 2013;13(10):723–32. doi: 10.1089/vbz.2012.1176 23919609

[26] Kwan JL, Kluh S, Madon MB, Nguyen DV, Barker CM, Reisen WK. Sentinel chicken seroconversions track tangential transmission of West Nile virus to humans in the greater Los Angeles area of California. Am J Trop Med Hyg. 2010;83(5):1137–45. 21036853 10.4269/ajtmh.2010.10-0078 PMC2963985

[27] Chevalier V, Lancelot R, Diaïte A, Mondet B, De Lamballerie X. Use of sentinel chickens to study the transmission dynamics of West Nile virus in a sahelian ecosystem. Epidemiol Infect. 2008;136(4):525–8. doi: 10.1017/S0950268807008801 17559695 PMC2870832

[28] Streng K, Atama N, Chandler F, Blom R, van der Jeugd H, Schrama M, et al. Sentinel chicken surveillance reveals previously undetected circulation of West Nile virus in the Netherlands. Emerg Microbes Infect. 2024;13(1):2406278. doi: 10.1080/22221751.2024.2406278 39295515 PMC11441057

[29] Amdouni J, Monaco F, Portanti O, Sghaier S, Conte A, Hassine TB, et al. Detection of enzootic circulation of a new strain of West Nile virus lineage 1 in sentinel chickens in the north of Tunisia. Acta Trop. 2020;202:105223. doi: 10.1016/j.actatropica.2019.105223 31647898

[30] Fall AG, Diaïté A, Seck MT, Bouyer J, Lefrançois T, Vachiéry N, et al. West Nile virus transmission in sentinel chickens and potential mosquito vectors, Senegal River Delta, 2008-2009. Int J Environ Res Public Health. 2013;10(10):4718–27. doi: 10.3390/ijerph10104718 24084679 PMC3823322

[31] Chaintoutis SC, Dovas CI, Papanastassopoulou M, Gewehr S, Danis K, Beck C. Evaluation of a West Nile virus surveillance and early warning system in Greece, based on domestic pigeons. Comp Immunol Microbiol Infect Dis. 2014;37(2):131–41. 24503179 10.1016/j.cimid.2014.01.004

[32] Caballero-Gómez J, Cano-Terriza D, Lecollinet S, Carbonell MD, Martínez-Valverde R, Martínez-Nevado E. Evidence of exposure to zoonotic flaviviruses in zoo mammals in Spain and their potential role as sentinel species. Vet Microbiol. 2020;247:108763. 32768215 10.1016/j.vetmic.2020.108763

[33] Kvapil P, Račnik J, Kastelic M, Bártová E, Korva M, Jelovšek M, et al. A Sentinel Serological Study in Selected Zoo Animals to Assess Early Detection of West Nile and Usutu Virus Circulation in Slovenia. Viruses. 2021;13(4):626. doi: 10.3390/v13040626 33917545 PMC8067518

[34] Hernandez-Colina A, Seechurn N, Costa T, Lopez J, Baylis M, Hesson JC. Surveillance of Culex spp. vectors and zoonotic arboviruses at a zoo in the United Kingdom. Heliyon. 2024;10(4):e26477. doi: 10.1016/j.heliyon.2024.e26477 PMC10884501 38404807

[35] Kilpatrick AM, Pape WJ. Predicting human West Nile virus infections with mosquito surveillance data. Am J Epidemiol. 2013;178(5):829–35. doi: 10.1093/aje/kwt046 23825164 PMC3755645

[36] Hadfield J, Brito AF, Swetnam DM, Vogels CBF, Tokarz RE, Andersen KG, et al. Twenty years of West Nile virus spread and evolution in the Americas visualized by Nextstrain. PLoS Pathog. 2019;15(10):e1008042. doi: 10.1371/journal.ppat.1008042 31671157 PMC6822705

[37] Petersen LR, Hayes EB. West Nile Virus in the Americas. Med Clin North Am. 2008;92(6):1307–22. 19145778 10.1016/j.mcna.2008.07.004

[38] Quirin R, Salas M, Zientara S, Zeller H, Labie J, Murri S. West Nile Virus, Guadeloupe. Emerg Infect Dis. 2004;10(4):706–8. 15200864 10.3201/eid1004.030465 PMC3323088

[39] Geffroy M, Pagès N, Chavernac D, Dereeper A, Aubert L, Herrmann-Storck C, et al. Shifting From Sectoral to Integrated Surveillance by Changing Collaborative Practices: Application to West Nile Virus Surveillance in a Small Island State of the Caribbean. Front Public Health. 2021;9:649190. doi: 10.3389/fpubh.2021.649190 34178915 PMC8222804

[40] WOAH. Event report 5739 - Guadeloupe, West Nile Fever. World Organisation for Animal Health. 2024. https://wahis.woah.org/#/in-event/5739/dashboard

[41] ARS Guadeloupe. Un premier cas humain d’infection par le virus West Nile (VWN) est détecté en Guadeloupe. 2024. https://www.guadeloupe.ars.sante.fr/media/128154/download?inline 2025 January 27.

[42] Lefrançois T, Blitvich BJ, Pradel J, Molia S, Vachiery N, Martinez D. West Nile virus in Guadeloupe: introduction, spread, and decrease in circulation level: 2002-2005. Ann N Y Acad Sci. 2006. 10.1196/annals.1373.025 17135513

[43] Ladreyt H, Auerswald H, Tum S, Ken S, Heng L, In S, et al. Comparison of Japanese Encephalitis Force of Infection in Pigs, Poultry and Dogs in Cambodian Villages. Pathogens. 2020;9(9):719. doi: 10.3390/pathogens9090719 32882890 PMC7558861

[44] Glennon EE, Becker DJ, Peel AJ, Garnier R, Suu-Ire RD, Gibson L. What is stirring in the reservoir? Modelling mechanisms of henipavirus circulation in fruit bat hosts. Philos Trans R Soc B Biol Sci. 2019;374(1782):20190021. 10.1098/rstb.2019.0021 PMC6711305 31401962

[45] Cauchemez S, Hoze N, Cousien A, Nikolay B, Ten Bosch Q. How Modelling Can Enhance the Analysis of Imperfect Epidemic Data. Trends Parasitol. 2019;35(5):369–79. doi: 10.1016/j.pt.2019.01.009 30738632 PMC7106457

[46] Yman V, White MT, Rono J, Arcà B, Osier FH, Troye-Blomberg M. Antibody acquisition models: A new tool for serological surveillance of malaria transmission intensity. Sci Rep. 2016;6(1):19472. doi: 10.1038/srep19472 26846726 PMC4984902

[47] Hozé N, Salje H, Rousset D, Fritzell C, Vanhomwegen J, Bailly S, et al. Reconstructing Mayaro virus circulation in French Guiana shows frequent spillovers. Nat Commun. 2020;11(1):2842. doi: 10.1038/s41467-020-16516-x 32503971 PMC7275077

[48] Hozé N, Diarra I, Sangaré AK, Pastorino B, Pezzi L, Kouriba B, et al. Model-based assessment of Chikungunya and O’nyong-nyong virus circulation in Mali in a serological cross-reactivity context. Nat Commun. 2021;12(1):6735. doi: 10.1038/s41467-021-26707-9 34795213 PMC8602252

[49] Diarra I, Nurtop E, Sangaré AK, Sagara I, Pastorino B, Sacko S. Zika Virus Circulation in Mali. Emerg Infect Dis. 2020;26(5):945–52. 32310065 10.3201/eid2605.191383 PMC7181926

[50] Duque MP, Naser AM, dos Santos GR, O’Driscoll M, Paul KK, Rahman M. Informing an investment case for Japanese encephalitis vaccine introduction in Bangladesh. Sci Adv. 2024;10(32):eadp1657. 10.1126/sciadv.adp1657 PMC11313847 39121225

[51] Nemoto T, Aubry M, Teissier Y, Paul R, Cao-Lormeau V-M, Salje H, et al. Reconstructing long-term dengue virus immunity in French Polynesia. PLoS Negl Trop Dis. 2022;16(10):e0010367. doi: 10.1371/journal.pntd.0010367 36191046 PMC9560594

[52] Kang H, Auzenbergs M, Clapham H, Maure C, Kim JH, Salje H, et al. Chikungunya seroprevalence, force of infection, and prevalence of chronic disability after infection in endemic and epidemic settings: a systematic review, meta-analysis, and modelling study. Lancet Infect Dis. 2024;24(5):488–503. 38342105 10.1016/S1473-3099(23)00810-1

[53] Ribeiro dos Santos G, Buddhari D, Iamsirithaworn S, Khampaen D, Ponlawat A, Fansiri T. Individual, household, and community drivers of dengue virus infection risk in Kamphaeng Phet Province, Thailand. J Infect Dis. 2022;226(8):1348–56. 35512137 10.1093/infdis/jiac177 PMC9574660

[54] Durand B, Balança G, Baldet T, Chevalier V. A metapopulation model to simulate West Nile virus circulation in Western Africa, Southern Europe and the Mediterranean basin. Vet Res. 2010;41(3):32. doi: 10.1051/vetres/2010004 20167194 PMC2826092

[55] Ferraguti M, Heesterbeek H, Martínez-de la Puente J, Jiménez-Clavero MÁ, Vázquez A, Ruiz S, et al. The role of different Culex mosquito species in the transmission of West Nile virus and avian malaria parasites in Mediterranean areas. Transbound Emerg Dis. 2021;68(2):920–30. doi: 10.1111/tbed.13760 32748497

[56] Hartley DM, Barker CM, Menach AL, Niu T, Gaff HD, Reisen WK. Effects of Temperature on Emergence and Seasonality of West Nile Virus in California. 2012 May 1 https://www.ajtmh.org/view/journals/tpmd/86/5/article-p884.xml 10.4269/ajtmh.2012.11-0342 PMC3335698 22556092

[57] Pradel J, Chalvet Monfray K, Molia S, Vachiéry N, Rousteau A, Imbert D. Risk factors for West Nile virus seropositivity of equids in Guadeloupe. Prev Vet Med. 2009;92(1):71–8. 19664833 10.1016/j.prevetmed.2009.07.001

[58] Beck C, Leparc Goffart I, Franke F, Gonzalez G, Dumarest M, Lowenski S, et al. Contrasted Epidemiological Patterns of West Nile Virus Lineages 1 and 2 Infections in France from 2015 to 2019. Pathogens. 2020;9(11):908. doi: 10.3390/pathogens9110908 33143300 PMC7692118

[59] Beck C, Lowenski S, Durand B, Bahuon C, Zientara S, Lecollinet S. Improved reliability of serological tools for the diagnosis of West Nile fever in horses within Europe. PLoS Negl Trop Dis. 2017;11(9):e0005936. doi: 10.1371/journal.pntd.0005936 28915240 PMC5617233

[60] Blitvich BJ, Bowen RA, Marlenee NL, Hall RA, Bunning ML, Beaty BJ. Epitope-Blocking Enzyme-Linked Immunosorbent Assays for Detection of West Nile Virus Antibodies in Domestic Mammals. J Clin Microbiol. 2003;41(6):2676–9. 12791902 10.1128/JCM.41.6.2676-2679.2003 PMC156482

[61] Pages N, Vachiéry N, Lefrançois T, Giraud-Girard K, Albina E, Pradel J. West-Nile virus surveillance in Guadeloupe, French West Indies. New technology conquering old vectors?. Palma de Mallorca, Spain: SOVE. 2017.

[62] Plummer M, Stukalov A, Denwood M, Plummer MM. Package ‘rjags’. Austria: Vienna. 2016.

[63] Hogrefe WR, Moore R, Lape-Nixon M, Wagner M, Prince HE. Performance of immunoglobulin G (IgG) and IgM enzyme-linked immunosorbent assays using a West Nile virus recombinant antigen (preM/E) for detection of West Nile virus- and other flavivirus-specific antibodies. J Clin Microbiol. 2004;42(10):4641–8. doi: 10.1128/JCM.42.10.4641-4648.2004 15472323 PMC522294

[64] Girl P, Euringer K, Coroian M, Mihalca AD, Borde JP, Dobler G. Comparison of Five Serological Methods for the Detection of West Nile Virus Antibodies. Viruses. 2024;16(5):788. doi: 10.3390/v16050788 38793670 PMC11126072

[65] Manual of Diagnostic Tests and Vaccines for Terrestrial Animals. World Organisation for Animal Health. 2024. https://www.woah.org/en/what-we-do/standards/codes-and-manuals/terrestrial-manual-online-access/

[66] Spiegelhalter DJ, Best NG, Carlin BP, Van Der Linde A. Bayesian measures of model complexity and fit. J R Stat Soc Ser B Stat Methodol. 2002;64(4):583–639.

[67] Roiz D, Ruiz S, Soriguer R, Figuerola J. Climatic effects on mosquito abundance in Mediterranean wetlands. Parasit Vectors. 2014;7(1):333. 25030527 10.1186/1756-3305-7-333 PMC4223583

[68] Poh KC, Chaves LF, Reyna-Nava M, Roberts CM, Fredregill C, Bueno R. The influence of weather and weather variability on mosquito abundance and infection with West Nile virus in Harris County, Texas, USA. Sci Total Environ. 2019;675:260–72. 31030133 10.1016/j.scitotenv.2019.04.109

[69] Marini G, Calzolari M, Angelini P, Bellini R, Bellini S, Bolzoni L, et al. A quantitative comparison of West Nile virus incidence from 2013 to 2018 in Emilia-Romagna, Italy. PLoS Negl Trop Dis. 2020;14(1):e0007953. doi: 10.1371/journal.pntd.0007953 31895933 PMC6939904

[70] Fairbanks EL, Daly JM, Tildesley MJ. Modelling the influence of climate and vector control interventions on arbovirus transmission. Viruses. 2024;16(8):1221. 39205195 10.3390/v16081221 PMC11359451

[71] Camp JV, Nowotny N. The knowns and unknowns of West Nile virus in Europe: what did we learn from the 2018 outbreak?. Expert Rev Anti Infect Ther. 2020;18(2):145–54. doi: 10.1080/14787210.2020.1713751 31914833

[72] DeFelice NB, Little E, Campbell SR, Shaman J. Ensemble forecast of human West Nile virus cases and mosquito infection rates. Nat Commun. 2017;8:14592. doi: 10.1038/ncomms14592 28233783 PMC5333106

[73] Calzolari M, Pautasso A, Montarsi F, Albieri A, Bellini R, Bonilauri P, et al. West Nile Virus Surveillance in 2013 via Mosquito Screening in Northern Italy and the Influence of Weather on Virus Circulation. PLoS One. 2015;10(10):e0140915. doi: 10.1371/journal.pone.0140915 26488475 PMC4619062

[74] Liu A, Lee V, Galusha D, Slade MD, Diuk-Wasser M, Andreadis T. Risk factors for human infection with West Nile virus in Connecticut: a multi-year analysis. Int J Health Geogr. 2009;8(1):67. 19943935 10.1186/1476-072X-8-67 PMC2788533

[75] Kwan JL, Kluh S, Reisen WK. Antecedent avian immunity limits tangential transmission of West Nile virus to humans. PLoS One. 2012;7(3):e34127. doi: 10.1371/journal.pone.0034127 22457819 PMC3311586

[76] Mencattelli G, Ndione MHD, Silverj A, Diagne MM, Curini V, Teodori L, et al. Spatial and temporal dynamics of West Nile virus between Africa and Europe. Nat Commun. 2023;14(1):6440. doi: 10.1038/s41467-023-42185-7 37833275 PMC10575862

[77] Meyer Steiger DB, Ritchie SA, Laurance SGW. Mosquito communities and disease risk influenced by land use change and seasonality in the Australian tropics. Parasit Vectors. 2016;9(1):387. doi: 10.1186/s13071-016-1675-2 27388293 PMC4936001

[78] Kishimoto-Yamada K, Itioka T. How much have we learned about seasonality in tropical insect abundance since Wolda (1988)?. Entomol Sci. 2015;18(4):407–19.

[79] Hancock C, Camp JV. Habitat-specific host selection patterns of Culex quinquefasciatus and Culex nigripalpus in Florida. J Am Mosq Control Assoc. 2022;38(2):83–91. 35588178 10.2987/21-7054

[80] Anderson JF, Fish D, Armstrong PM, Misencik MJ, Bransfield A, Ferrandino FJ. Seasonal dynamics of mosquito-borne viruses in the southwestern Florida Everglades, 2016, 2017. Am J Trop Med Hyg. 2022;106(2):610–22. 35008051 10.4269/ajtmh.20-1547 PMC8832897

[81] Hamer GL, Walker ED, Brawn JD, Loss SR, Ruiz MO, Goldberg TL, et al. Rapid amplification of West Nile virus: the role of hatch-year birds. Vector Borne Zoonotic Dis. 2008;8(1):57–67. doi: 10.1089/vbz.2007.0123 18237262

[82] Cañizares JR, Edwards CB, Reed JM. Quantifying phenological landmarks of migration shows nonuniform use of the Caribbean by shorebirds. Ecol Evol. 2023;13(4):e9954. doi: 10.1002/ece3.9954 37038523 PMC10082156

[83] Cissé B, Lapen DR, Chalvet-Monfray K, Ogden NH, Ludwig A. Modeling West Nile Virus transmission in birds and humans: Advantages of using a cellular automata approach. Infect Dis Model. 2024;9(1):278–97. 38328278 10.1016/j.idm.2024.01.002 PMC10847944

[84] Bergsman LD, Hyman JM, Manore CA. A mathematical model for the spread of west nile virus in migratory and resident birds. Math Biosci Eng. 2016;13(2):401–24. doi: 10.3934/mbe.2015009 27105987

[85] Swetnam D, Widen SG, Wood TG, Reyna M, Wilkerson L, Debboun M, et al. Terrestrial Bird Migration and West Nile Virus Circulation, United States. Emerg Infect Dis. 2018;24(12):2184–94. doi: 10.3201/eid2412.180382 30457531 PMC6256381

[86] Andreadis TG, Anderson JF, Vossbrinck CR, Main AJ. Epidemiology of West Nile virus in Connecticut: a five-year analysis of mosquito data 1999-2003. Vector Borne Zoonotic Dis. 2004;4(4):360–78. doi: 10.1089/vbz.2004.4.360 15682518

[87] Giordano BV, Kaur S, Hunter FF. West Nile virus in Ontario, Canada: A twelve-year analysis of human case prevalence, mosquito surveillance, and climate data. PLoS One. 2017;12(8):e0183568. doi: 10.1371/journal.pone.0183568 28829827 PMC5568768

[88] Wilcox BR, Yabsley MJ, Ellis AE, Stallknecht DE, Gibbs SEJ. West Nile virus antibody prevalence in American crows (Corvus brachyrhynchos) and fish crows (Corvus ossifragus) in Georgia, USA. Avian Dis. 2007;51(1):125–8. doi: 10.1637/0005-2086(2007)051[0125:WNVAPI]2.0.CO;2 17461278

[89] Gibbs SEJ, Hoffman DM, Stark LM, Marlenee NL, Blitvich BJ, Beaty BJ, et al. Persistence of antibodies to West Nile virus in naturally infected rock pigeons (Columba livia). Clin Diagn Lab Immunol. 2005;12(5):665–7. doi: 10.1128/CDLI.12.5.665-667.2005 15879030 PMC1112075

[90] Nemeth NM, Kratz GE, Bates R, Scherpelz JA, Bowen RA, Komar N. Naturally induced humoral immunity to West Nile virus infection in raptors. EcoHealth. 2008;5(3):298–304. 18677535 10.1007/s10393-008-0183-z

[91] Nemeth NM, Oesterle PT, Bowen RA. Humoral Immunity to West Nile Virus Is Long-Lasting and Protective in the House Sparrow (Passer domesticus). 2009 May 1 https://www.ajtmh.org/view/journals/tpmd/80/5/article-p864.xml PMC2693945 19407139

[92] Chevalier V, Lecollinet S, Durand B. West Nile virus in Europe: a comparison of surveillance system designs in a changing epidemiological context. Vector Borne Zoonotic Dis. 2011;11(8):1085–91. doi: 10.1089/vbz.2010.0234 21548765

[93] Edman JD, Webber LA, Schmid AA. Effect of host defenses on the feeding pattern of Culex nigripalpus when offered a choice of blood sources. J Parasitol. 1974;60(5):874–83. 4430956

[94] Mackay AJ, Kramer WL, Meece JK, Brumfield RT, Foil LD. Host Feeding Patterns of Culex Mosquitoes (Diptera: Culicidae) in East Baton Rouge Parish, Louisiana. J Med Entomol. 2010;47(2):238–48. 20380306 10.1603/me09168

[95] Kay BH, Boreham PF, Fanning ID. Host-feeding patterns of Culex annulirostris and other mosquitoes (Diptera: Culicidae) at Charleville, southwestern Queensland, Australia. J Med Entomol. 1985;22(5):529–35. doi: 10.1093/jmedent/22.5.529 2864452

[96] Jansen CC, Webb CE, Graham GC, Craig SB, Zborowski P, Ritchie SA, et al. Blood Sources of Mosquitoes Collected from Urban and Peri-Urban Environments in Eastern Australia with Species-Specific Molecular Analysis of Avian Blood Meals. 2009 Nov 1 https://www.ajtmh.org/view/journals/tpmd/81/5/article-p849.xml 10.4269/ajtmh.2009.09-0008 19861621

[97] Boyer S, Durand B, Yean S, Brengues C, Maquart P-O, Fontenille D, et al. Host-Feeding Preference and Diel Activity of Mosquito Vectors of the Japanese Encephalitis Virus in Rural Cambodia. Pathogens. 2021;10(3):376. doi: 10.3390/pathogens10030376 33800999 PMC8003966

[98] Komar N, Clark GG. West Nile virus activity in Latin America and the Caribbean. Rev Panam Salud Publica. 2006;19(2):112–7. doi: 10.1590/s1020-49892006000200006 16551385

[99] Filière équine Antilles-Guyane - Observatoire Economique Régional. 2017. https://www.ifce.fr/wp-content/uploads/2017/02/OESC-OERV3-Antilles-Guyane.pdf

[100] Gardner IA, Wong SJ, Ferraro GL, Balasuriya UB, Hullinger PJ, Wilson WD, et al. Incidence and effects of West Nile virus infection in vaccinated and unvaccinated horses in California. Vet Res. 2007;38(1):109–16. doi: 10.1051/vetres:2006045 17274156

[101] Bunning ML, Bowen RA, Cropp CB, Sullivan KG, Davis BS, Komar N, et al. Experimental infection of horses with West Nile virus. Emerg Infect Dis. 2002;8(4):380–6. doi: 10.3201/eid0804.010239 11971771 PMC3393377

[102] L’Azou M, Taurel A-F, Flamand C, Quénel P. Recent epidemiological trends of dengue in the French territories of the Americas (2000-2012): a systematic literature review. PLoS Negl Trop Dis. 2014;8(11):e3235. doi: 10.1371/journal.pntd.0003235 25375627 PMC4222734

[103] Beck C, Leparc-Goffart I, Desoutter D, Debergé E, Bichet H, Lowenski S, et al. Serological evidence of infection with dengue and Zika viruses in horses on French Pacific Islands. PLoS Negl Trop Dis. 2019;13(2):e0007162. doi: 10.1371/journal.pntd.0007162 30730887 PMC6382171

[104] de Wit MM, Dimas Martins A, Delecroix C, Heesterbeek H, ten Bosch QA. Mechanistic models for West Nile virus transmission: a systematic review of features, aims and parametrization. Proc R Soc B Biol Sci. 2024;291(2018):20232432. 10.1098/rspb.2023.2432 PMC10932716 38471554

