# Supplementary material for: Reconstructing the silent circulation of West Nile Virus in a Caribbean island during 15 years using sentinel serological data
Source: PLoS Negl Trop Dis. 2025 Jun 23;19(6):e0012895. doi: 10.1371/journal.pntd.0012895 (PMC12212876; doi:10.1371/journal.pntd.0012895)
Supplement: S2 Fig — (PDF) [file pntd.0012895.s002.pdf]

S2 Fig

Reconstructing the silent circulation of West Nile Virus in a Caribbean island during 15 years using sentinel serological data

Celia Hamouche, Jennifer Pradel, Nonito Pagès, Véronique Chevalier, Sylvie Lecollinet, Jonathan Bastard *, Benoit Durand *

* These authors contributed equally to this work.

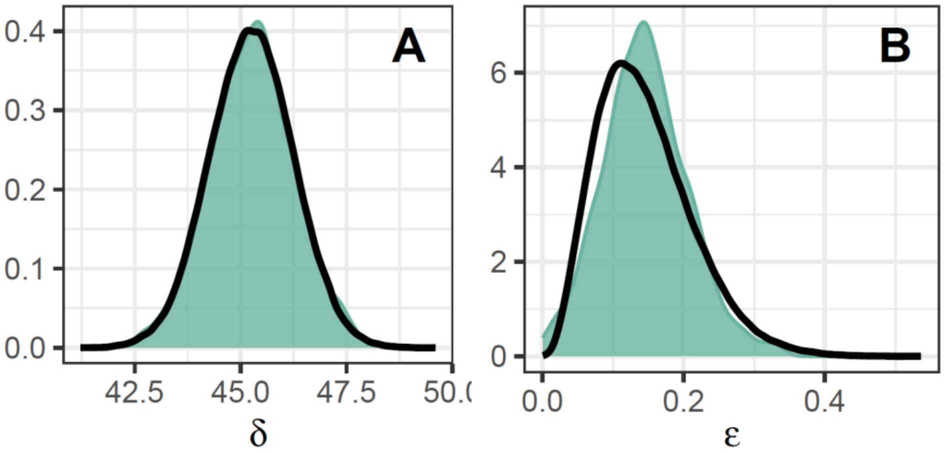

S2 Fig. Panel A: posterior distribution of δ following Step 1 (colored area), and corresponding Normal distribution (of mean 45.26 and standard deviation 0.988) used as prior distribution in Step 2 (black line). Panel B: posterior distribution of ε following Step 1 (colored area), and corresponding Beta distribution (of parameters 3.80 and 22.43) used as prior distribution in Step 2 (black line).
