# Supplementary material for: Reconstructing the silent circulation of West Nile Virus in a Caribbean island during 15 years using sentinel serological data
Source: PLoS Negl Trop Dis. 2025 Jun 23;19(6):e0012895. doi: 10.1371/journal.pntd.0012895 (PMC12212876; doi:10.1371/journal.pntd.0012895)
Supplement: S3 Fig — The base layer map for this figure was obtained from GADM: https://gadm.org/download_country.html (link to the license information: https://gadm.org/license.html (PDF) [file pntd.0012895.s003.pdf]

S3 Fig

Reconstructing the silent circulation of West Nile Virus in a Caribbean island during 15 years using sentinel serological data

Celia Hamouche, Jennifer Pradel, Nonito Pagès, Véronique Chevalier, Sylvie Lecollinet, Jonathan Bastard *, Benoit Durand *

* These authors contributed equally to this work.

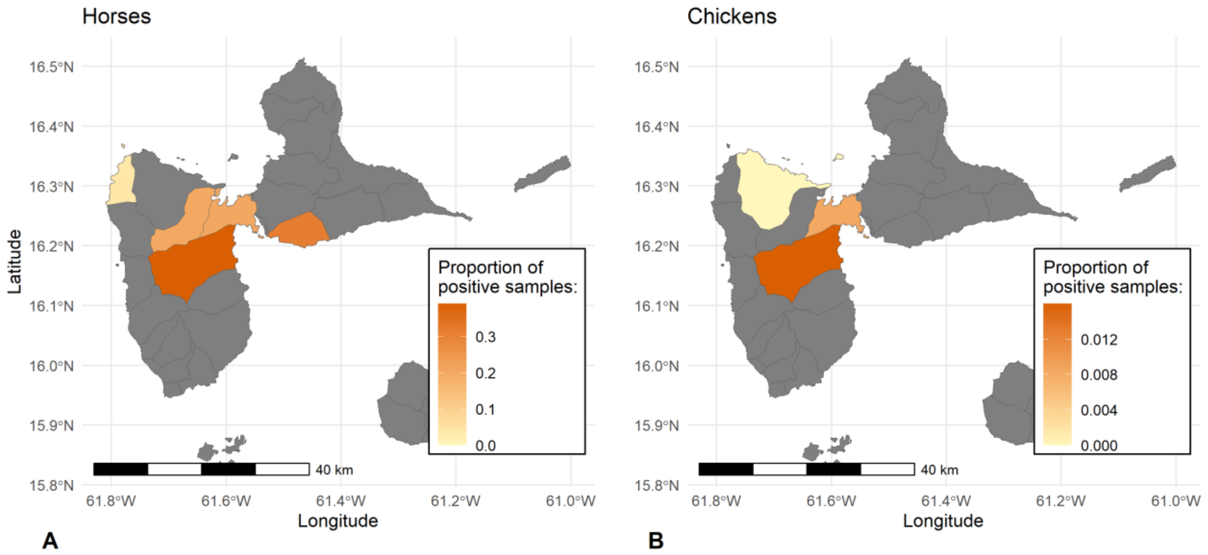

S3 Fig. Map with proportion of collected samples that were positive to anti-WNV IgG antibodies per commune, in horses (panel A) and chickens (panel B). The model analyzed serological transitions between pairs of samples, rather than raw serological results. The base layer map for this figure was obtained from GADM: https://gadm.org/download_country.html (link to the license information: https://gadm.org/license.html).
