# Supplementary material for: Reconstructing the silent circulation of West Nile Virus in a Caribbean island during 15 years using sentinel serological data
Source: PLoS Negl Trop Dis. 2025 Jun 23;19(6):e0012895. doi: 10.1371/journal.pntd.0012895 (PMC12212876; doi:10.1371/journal.pntd.0012895)
Supplement: S4 Fig — (PDF) [file pntd.0012895.s004.pdf]

# S4 Fig

# Reconstructing the silent circulation of West Nile Virus in a Caribbean island during 15 years using sentinel serological data

Celia Hamouche, Jennifer Pradel, Nonito Pagès, Véronique Chevalier, Sylvie Lecollinet, Jonathan Bastard *, Benoit Durand *

* These authors contributed equally to this work.

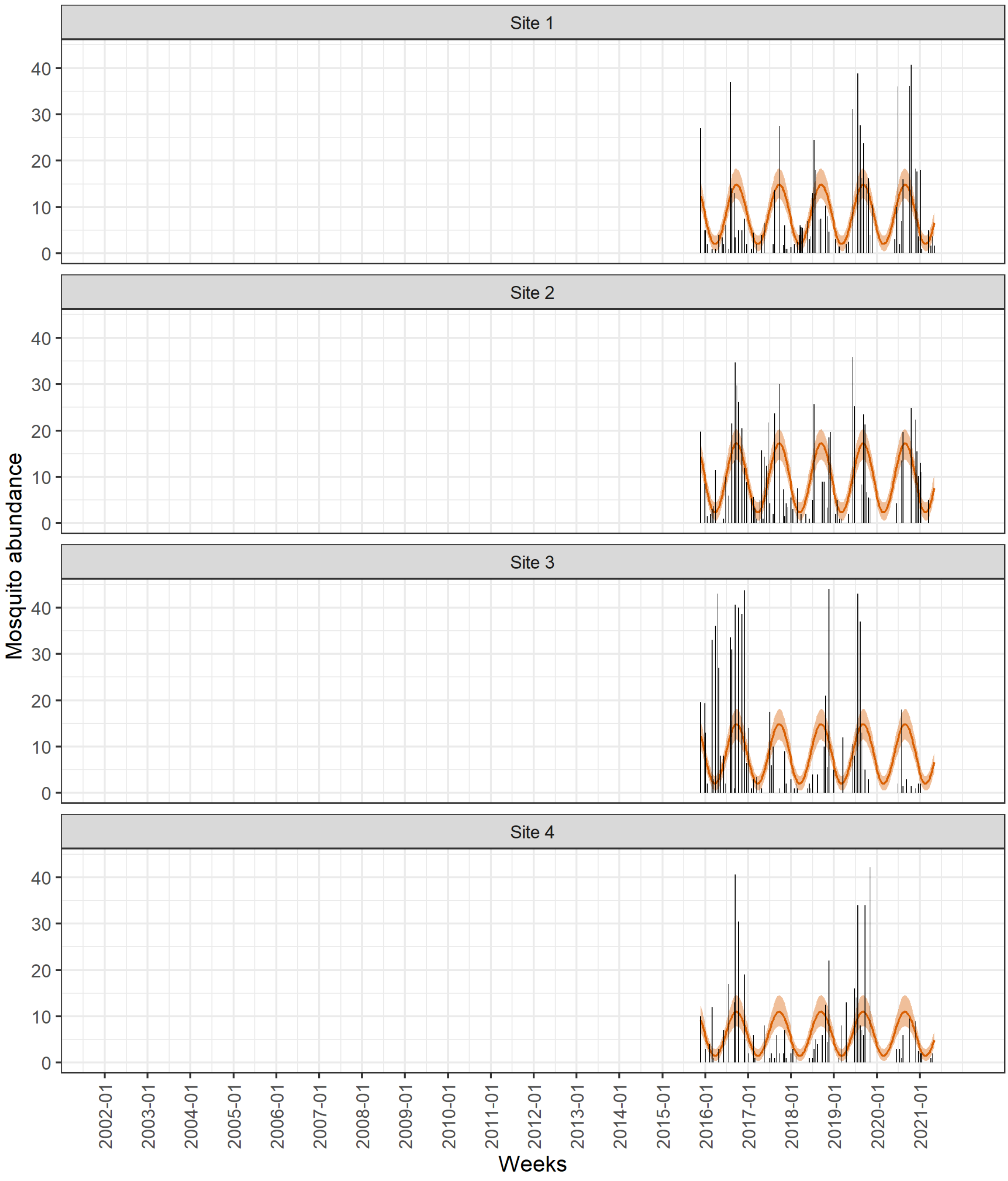

**S4 Fig.** Mosquito abundance variations in four collection sites in Guadeloupe between November 2015 and May 2021: observed data (vertical bars) and model predictions following Step 1 (median of 500 model repetitions: solid line; 95% prediction interval: colored area). The x-axis scale (2002-2021) is standardized with the serological sampling periods (Figure 2 in the main manuscript).
