# Supplementary material for: Reconstructing the silent circulation of West Nile Virus in a Caribbean island during 15 years using sentinel serological data
Source: PLoS Negl Trop Dis. 2025 Jun 23;19(6):e0012895. doi: 10.1371/journal.pntd.0012895 (PMC12212876; doi:10.1371/journal.pntd.0012895)
Supplement: S5 Fig — (PDF) [file pntd.0012895.s005.pdf]

S5 Fig

Reconstructing the silent circulation of West Nile Virus in a Caribbean island during 15 years using sentinel serological data

Celia Hamouche, Jennifer Pradel, Nonito Pagès, Véronique Chevalier, Sylvie Lecollinet, Jonathan Bastard *, Benoit Durand *

* These authors contributed equally to this work.

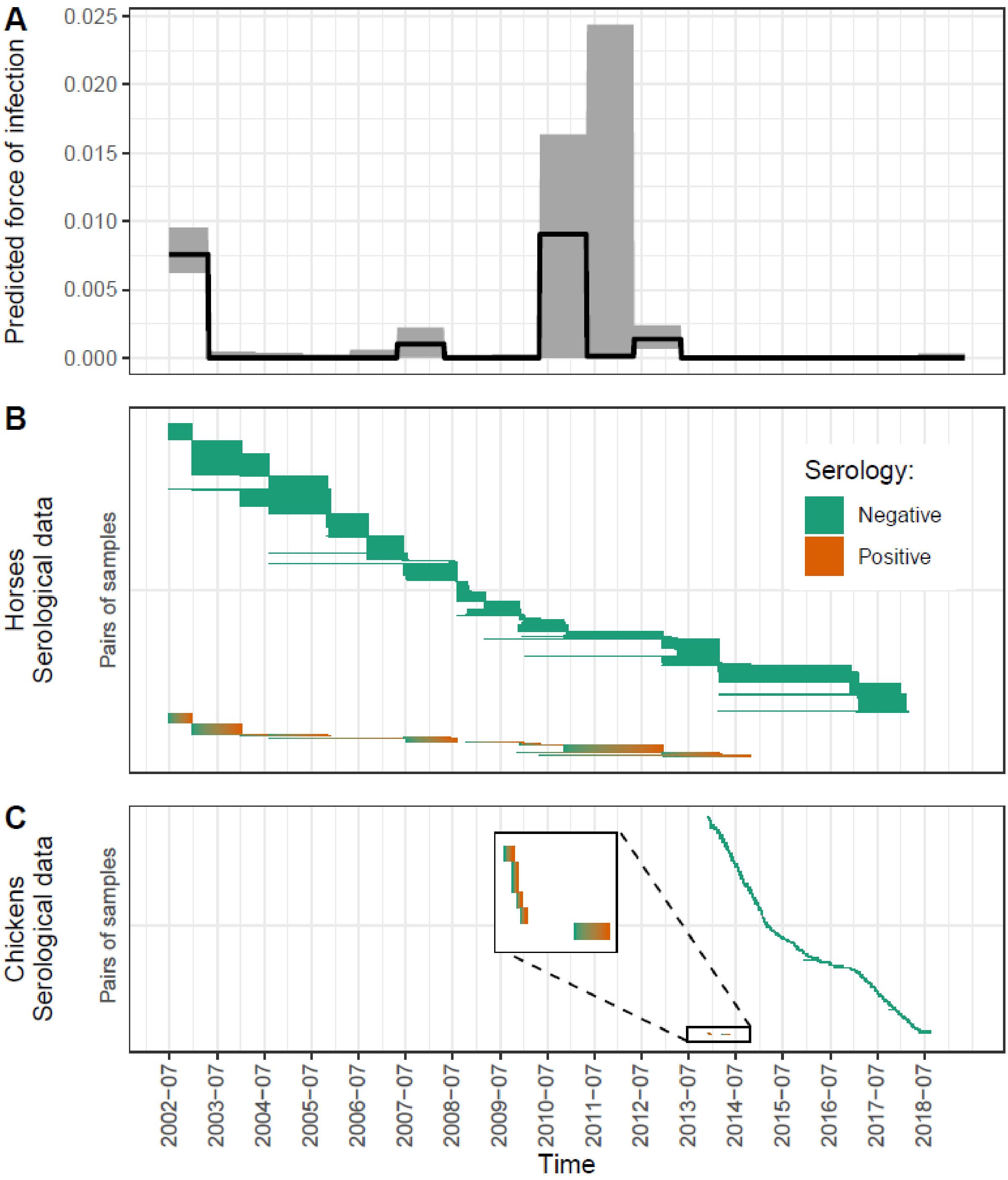

S5 Fig. WNV force of infection (FOI) in Guadeloupe predicted between 2002 and 2017 by the "FlatVary" serological model (panel A), and longitudinal serological data collected in horses (panel B) and chickens (panel C). In panel A, the black line represents the median of predictions (using 5,000 repetitions of the model), while the gray area represents the 80% prediction interval. In panels B and C, each row is a pair of consecutive blood samples, and only the observed negative-to-positive and negative-to-negative serological transitions are displayed.
