# Supplementary material for: Reconstructing the silent circulation of West Nile Virus in a Caribbean island during 15 years using sentinel serological data
Source: PLoS Negl Trop Dis. 2025 Jun 23;19(6):e0012895. doi: 10.1371/journal.pntd.0012895 (PMC12212876; doi:10.1371/journal.pntd.0012895)
Supplement: S7 Fig — (PDF) [file pntd.0012895.s007.pdf]

S7 Fig

Reconstructing the silent circulation of West Nile Virus in a Caribbean island during 15 years using sentinel serological data

Celia Hamouche, Jennifer Pradel, Nonito Pagès, Véronique Chevalier, Sylvie Lecollinet, Jonathan Bastard *, Benoit Durand *

* These authors contributed equally to this work.

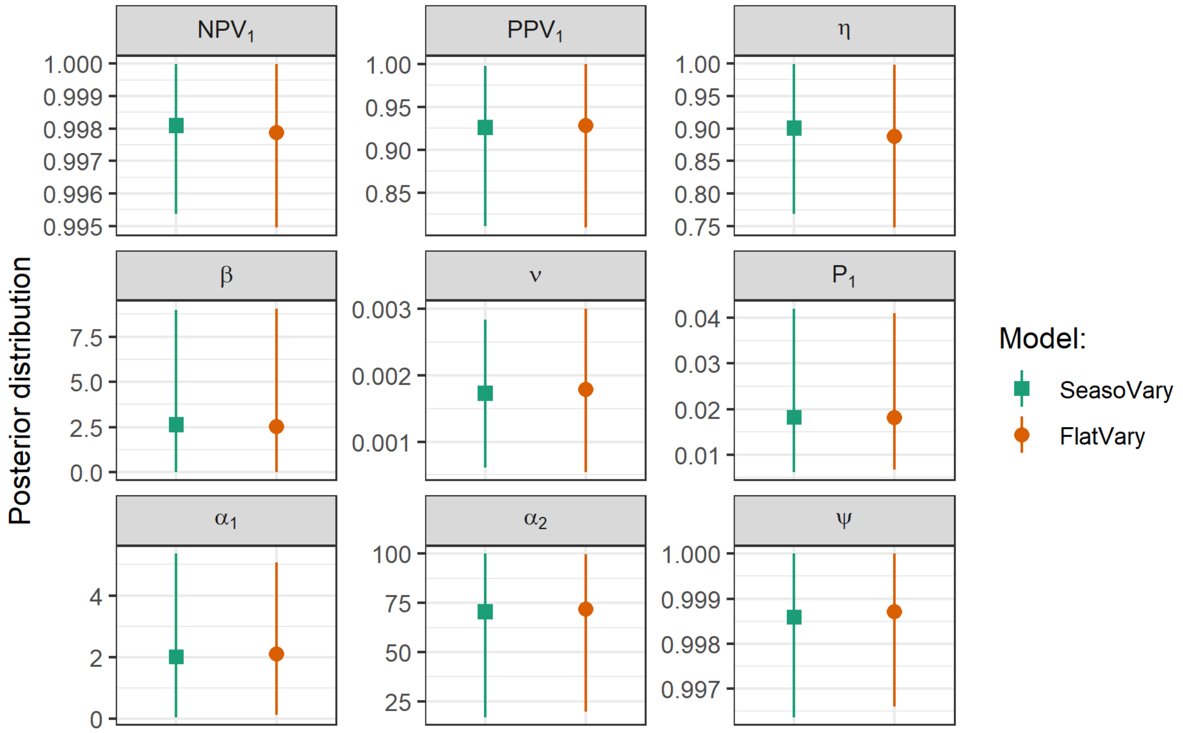

S7 Fig. Comparison of the parameters' posterior distributions with the "FlatVary" and "SeasoVary" models. Some parameters of the "SeasoVary" model are not used in the "FlatVary" model (see Table 1 of the main manuscript).
