# Supplementary material for: Reconstructing the silent circulation of West Nile Virus in a Caribbean island during 15 years using sentinel serological data
Source: PLoS Negl Trop Dis. 2025 Jun 23;19(6):e0012895. doi: 10.1371/journal.pntd.0012895 (PMC12212876; doi:10.1371/journal.pntd.0012895)
Supplement: S1 Table — (PDF) [file pntd.0012895.s008.pdf]

S1 Table

Reconstructing the silent circulation of West Nile Virus in a Caribbean island during 15 years using sentinel serological data

Celia Hamouche, Jennifer Pradel, Nonito Pagès, Véronique Chevalier, Sylvie Lecollinet, Jonathan Bastard *, Benoit Durand *

* These authors contributed equally to this work.

S1 Table. Formula of the force of infection depending on the serological model scenario and the species.

**Model scenario**	**Species**	**Force of infection *λ(t)***
**FlatStable**	Horses	Λ
	Chickens	$\beta.\Lambda$
**FlatVary**	Horses	$\Lambda(y(t))$
	Chickens	$\beta.\Lambda(y(t))$
**SeasoStable**	Horses	$\frac{\Lambda}{2}(1-\varepsilon)\left(1+cos\left(\frac{2\pi}{52}\ (t-\delta)\right)\right)+\varepsilon.\Lambda$
	Chickens	$\beta\left[\frac{\Lambda}{2}(1-\varepsilon)\left(1+cos\left(\frac{2\pi}{52}\ (t-\delta)\right)\right)+\varepsilon.\Lambda\right]$
**SeasoVary**	Horses	$\frac{\Lambda(y(t))}{2}(1-\varepsilon)\left(1+cos\left(\frac{2\pi}{52}\ (t-\delta)\right)\right)+\varepsilon.\Lambda(y(t))$
	Chickens	$\beta\left[\frac{\Lambda(y(t))}{2}(1-\varepsilon)\left(1+cos\left(\frac{2\pi}{52}\ (t-\delta)\right)\right)+\varepsilon.\Lambda(y(t))\right]$
