# Supplementary material for: Reconstructing the silent circulation of West Nile Virus in a Caribbean island during 15 years using sentinel serological data
Source: PLoS Negl Trop Dis. 2025 Jun 23;19(6):e0012895. doi: 10.1371/journal.pntd.0012895 (PMC12212876; doi:10.1371/journal.pntd.0012895)
Supplement: S2 Table — (PDF) [file pntd.0012895.s009.pdf]

S2 Table

Reconstructing the silent circulation of West Nile Virus in a Caribbean island during 15 years using sentinel serological data

Celia Hamouche, Jennifer Pradel, Nonito Pagès, Véronique Chevalier, Sylvie Lecollinet, Jonathan Bastard *, Benoit Durand *

* These authors contributed equally to this work.

S2 Table. Prior distributions used in Steps 1 and 2 of model fitting. Posterior distributions estimated for ε and δ following Step 1 (i.e. the fit of the seasonal model to mosquito abundance data) were then used as informative Prior distributions for Step 2 (i.e. the fit of all models to serological data).

Parameter	Prior in Step 1<br>Fit to mosquito abundance data	Prior in Step 2<br>Fit to serological data
β	-	Uniform(0, 10)
$\Lambda(y(t))$ or Λ	-	Exponential transformation of Uniform(-20, 3)
ε	Uniform(0, 1)	Beta(3.80, 22.43)
δ	Uniform(1, 53)	Normal(45.26, 0.988)
μ	-	Uniform(0, 0.5)
η	-	Beta(3, 1)
Ψ	-	Beta(3, 1)
NPV_1	-	Depends on η, Ψ, α_1 and α_2 (see S1 Note)
PPV_1	-	Depends on η, Ψ, α_1 and α_2 (see S1 Note)
P_1	-	Depends on α_1 and α_2 (see S1 Note)
α_1	-	Uniform(0, 100)
α_2	-	Uniform(0, 100)
σ_{mosq}	Uniform(0, 100)	-
N_0	Exponential transformation of Uniform(-5, 10)	-
F_j	Uniform(0, 100)	-
