# Supplementary material for: Reconstructing the silent circulation of West Nile Virus in a Caribbean island during 15 years using sentinel serological data
Source: PLoS Negl Trop Dis. 2025 Jun 23;19(6):e0012895. doi: 10.1371/journal.pntd.0012895 (PMC12212876; doi:10.1371/journal.pntd.0012895)
Supplement: S3 Table — (PDF) [file pntd.0012895.s010.pdf]

S3 Table

Reconstructing the silent circulation of West Nile Virus in a Caribbean island during 15 years using sentinel serological data

Celia Hamouche, Jennifer Pradel, Nonito Pagès, Véronique Chevalier, Sylvie Lecollinet, Jonathan Bastard *, Benoit Durand *

* These authors contributed equally to this work.

S3 Table. Values of the Deviance Information Criterion (DIC) for the different serological model scenarios.

**Model scenario**	FlatStable	FlatVary	SeasoStable	SeasoVary
**DIC**	1083	935	1072	923
