# Supplementary material for: Reconstructing the silent circulation of West Nile Virus in a Caribbean island during 15 years using sentinel serological data
Source: PLoS Negl Trop Dis. 2025 Jun 23;19(6):e0012895. doi: 10.1371/journal.pntd.0012895 (PMC12212876; doi:10.1371/journal.pntd.0012895)
Supplement: S1 Note — Definitions of parameters NPV1 and PPV1. (PDF) [file pntd.0012895.s011.pdf]

S1 Note

Reconstructing the silent circulation of West Nile Virus in a Caribbean island during 15 years using sentinel serological data

Celia Hamouche, Jennifer Pradel, Nonito Pagès, Véronique Chevalier, Sylvie Lecollinet, Jonathan Bastard *, Benoit Durand *

* These authors contributed equally to this work.

S1 Note. Definitions of parameters NPV_1 and PPV_1

To initialize the model and infer the true serological status of the first sample in each individual i, we introduced a parameter NPV_1, the negative predictive value of the first sample result for any individual (main manuscript, Table 1). By definition [1]:

$$NPV_1 = \frac{\psi.(1 - P_1)}{\psi.(1 - P_1) + (1 - \eta).P_1} \qquad (S1)$$

Where P_1 was a parameter varying between 0 and 1. It was assumed to follow a Beta distribution of estimated parameters α_1 and α_2:

$$P_1 \sim Beta(\alpha_1, \alpha_2) \qquad (S2)$$

Similarly, PPV_1, the positive predictive value of the first sample result for any individual, was defined as:

$$PPV_1 = \frac{\eta.P_1}{(1 - \psi).(1 - P_1) + \eta.P_1} \qquad (S3)$$

It may be noted that P_1, NPV_1 and PPV_1 were time-independent and defined for the whole study period. It might be considered as a limitation since the negative and positive predicted values of a test depend on the prevalence which, in our study, may vary according to the age of the animal at the first sampling (information that we did not have), the time of this sampling and the species. Here, we did not have the statistical power to directly account for these multiple sources of variability of P_1, nor the data regarding the age at first sampling. Instead, we allowed P_1 (and therefore NPV_1 and PPV_1) to be distributed with a large variance, by defining it with α_1 and α_2 that have uninformative priors. Moreover, P_1 does not necessarily, straightforwardly, equal the seroprevalence when individuals were first sampled, because of the longitudinal nature of the data. Indeed, the model likelihood is evaluated using all the data, and the true serological status inferred by the model in the later samplings for a given individual (that depends on parameters including η and Ψ) affects the true serological status inferred in the first sampling for that individual (that depends on NPV_1 and PPV_1, hence on P_1, η and Ψ). Therefore, it might not be adequate to *a priori* define P_1 based on the observed seropositivity rate in first samplings, or to *a posteriori* compare it to the observed seropositivity rate in first samplings. The former may also pose the issue of using the same data to both inform the prior of P_1 and evaluate the likelihood.

Bibliography

1. Monaghan TF, Rahman SN, Agudelo CW, Wein AJ, Lazar JM, Everaert K, et al. Foundational Statistical Principles in Medical Research: Sensitivity, Specificity, Positive Predictive Value, and Negative Predictive Value. Medicina (Mex). 2021 May;57(5):503.
