# Supplementary material for: Reconstructing the silent circulation of West Nile Virus in a Caribbean island during 15 years using sentinel serological data
Source: PLoS Negl Trop Dis. 2025 Jun 23;19(6):e0012895. doi: 10.1371/journal.pntd.0012895 (PMC12212876; doi:10.1371/journal.pntd.0012895)
Supplement: S2 Note — Details on the first step of the model fitting. (PDF) [file pntd.0012895.s012.pdf]

S2 Note

Reconstructing the silent circulation of West Nile Virus in a Caribbean island during 15 years using sentinel serological data

Celia Hamouche, Jennifer Pradel, Nonito Pagès, Véronique Chevalier, Sylvie Lecollinet, Jonathan Bastard *, Benoit Durand *

* These authors contributed equally to this work.

S2 Note. Details on the first step of the model fitting

Step 1 of the model fitting procedure consisted to fit a model analogous to the "SeasoStable" model to the mosquito abundance data, in order to obtain estimates of parameters δ and ε, which were used in turn as priors for Step 2. The abundance of mosquitoes trapped at a site j on a week t was modelled as:

$$N_{mosq,j}(t) \sim Normal\left(E_{mosq,j}(t), \sigma_{mosq}\right) \qquad (S4)$$

Where:

$$E_{mosq,j}(t) = N_0 F_j \left((1-\varepsilon)\frac{1}{2}\left(1 + cos\left(\frac{2\pi}{52}\,(t-\delta)\right)\right) + \varepsilon \right) \qquad (S5)$$

Where N_0 was a scale parameter, F_j was the fixed effect of site j (with Site 1 as reference, i.e. $F_1 = 1$), and δ and ε were as defined in the main manuscript. All parameters were estimated with mostly uninformative priors (S2 Table).
